# Does age delay gastric emptying? Ultrasound reveals altered dynamics in the aged population

**DOI:** 10.3389/fmed.2026.1786573

**Published:** 2026-03-04

**Authors:** Kai Yang, Tao Huang, Lina Yu, Yiyang Tang, Bijing Li, Gaofeng Zhao, Wenfei Long

**Affiliations:** 1 Department of Anesthesiology, The First Affiliated Hospital of Guangzhou University of Chinese Medicine, Guangzhou, Guangdong, China; 2 Department of Anesthesiology, Fengshun County Traditional Chinese Medicine Hospital of Guangdong Province, Meizhou, China; 3 Department of Anesthesiology, The Second Affiliated Hospital of Guangzhou University of Chinese Medicine, Guangzhou, Guangdong, China; 4 Department of Ultrasound, The Second Affiliated Hospital of Guangzhou University of Chinese Medicine, Guangzhou, Guangdong, China; 5 State Key Laboratory of Traditional Chinese Medicine Syndrome, Guangzhou, China

**Keywords:** gastric emptying rate, gastric ultrasound, older adult, preoperative fasting, rice-based meal

## Abstract

**Background:**

The current preoperative fasting guideline of American Society of Anesthesiologists for adults, including younger and older adults, is 8 h of fasting. However, differences in gastric emptying between younger and older adults remain unclear. To determine fluid emptying time and 4 h gastric emptying rate (4HGER) in younger and older adults by evaluating the gastric antrum using ultrasound after ingestion of a rice-based meal.

**Methods:**

A total of 60 participants were enrolled, including 30 older adults (aged 65–80 years; median age 71.5 years) and 30 younger adults (aged 18–64 years; median age 41.5 years) with BMI ranging from 18 to 30 kg/m^2^. Participants underwent gastric ultrasound measurements every 15 min during a 120 min period after drinking 300 mL of water and at seven time points during a 240 min period after ingesting a 550 kcal rice-based meal. Gastric antral cross-sectional area (CSA) was measured, gastric volume (GV) was calculated, and 4HGER was determined based on CSA changes. Additional measurements included blood glucose levels and hunger scores using a numeric rating scale (NRS).

**Results:**

No significant difference was observed in fluid emptying time between younger and older adults (median 45 min; *P* = 0.737). However, the mean 4HGER was significantly lower in older adults (35% ± 9%) compared to younger adults (51% ± 12%; *P* < 0.05). Age and BMI were significant predictors of gastric emptying, with older age and higher BMI associated with delayed solid gastric emptying. Blood glucose levels were higher in older adults postprandially, but hunger scores did not significantly differ between groups.

**Conclusion:**

Gastric emptying of solid food, as assessed by ultrasound, is significantly delayed in older adults compared to younger adults, while liquid emptying time remains unaffected. These findings highlight the need to reconsider current fasting guidelines for older adults to minimize perioperative complications such as aspiration and hypoglycemia. The results have important clinical relevance for optimizing preoperative fasting protocols in elderly patients undergoing anesthesia.

**Clinical Trial Registration:**

Clinicaltrials.gov identifier ChiCTR2100045201 (date of registration: 8th April, 2021).

## 1 Introduction

Pulmonary aspiration of gastric contents can cause severe complications and even death. The practice guidelines published by the American Society of Anesthesiologists (ASA) recommend 2 and 8 h of fasting after ingestion of fluids and solids, respectively, for adults regardless of age (1). However, gastric emptying (GE) is affected by factors such as calorie consumption, viscosity, processing method, total amount of food consumed (2–5), age, sex, and diabetes (6–9). Findings on the effects of age on GE are conflicting. Moore et al. (10) used the dual radioisotopic method and reported that the emptying of solids did not change with age, whereas the emptying of liquids was delayed in older adults. Meal time and age are significant covariates in GE (11). Recently, a study comparing gastric emptying rate (GER) in older adult participants with well-controlled type 2 diabetes mellitus, age- and body mass index (BMI)-matched older adults without diabetes, and younger adults without diabetes found that GER was lower in older adults without diabetes than in younger adults without diabetes (12). Horowitz et al. (6) reported a significant delay in liquid and solid emptying in older adults, using a scintillation camera. Wegener et al. (13) noted that compared with that in 21 younger controls (age: 33.5 years), GER was lower in 25 older participants (age: 81.7 years) after ingestion of a wheat-based meal.

Gastric emptying is typically assessed by evaluating the impact of different types of food, including juice, coffee, milk (14, 15), bread (16), and lasagne (12). Moreover, the practice guidelines were developed according to a Western diet. Cho et al. (17) demonstrated that the GE time was longer after ingestion of a rice-based meal than after ingestion of meat sandwiches and Italian food, indicating that the GE data obtained from Western countries may not be applicable to Far East Asian populations.

Ultrasound measurement of the gastric antrum has been used to evaluate the gastric volume (GV) in children, adults, pregnant women, and neonates (18–20). The aim of the present study was to determine differences in GER of rice-based meals between younger and older adults using gastric ultrasound. We hypothesized that GER of rice-based food would be lower in older adults than in younger adults.

## 2 Materials and methods

### 2.1 Participants

This study was approved by the Institutional Ethics Board of the Guangdong Hospital of Chinese Medicine, Guangzhou, China (approval number ZE2020-162-01), and written informed consent was obtained from all participants. The trial was registered prior to participant enrolment at clinicaltrials.gov (ChiCTR2100045201, 8th April 2021).

The inclusion criteria were age 18–80 years, ASA physical status I–II, and BMI 18–30 kg/m^2^. The exclusion criteria were diabetes mellitus, history of gastrointestinal or stomach surgery, hiatus hernia, gastroesophageal reflux disease, severe cardiovascular diseases, abnormal renal function, and current pregnancy.

This study was conducted between April 2021 and September 2021. Participants were separated into two groups: an older adult group comprising individuals aged 65–80 years and a younger adult comparison group comprising individuals aged 18–64 years.

### 2.2 Study design and procedures

This manuscript adheres to the STROBE guidelines. The scanning protocol was modified by a certified sonographer. The participants were examined by a sonographer with previous experience of over 100 gastric ultrasound assessments who was blinded to the age of the volunteers. A trained anasthesiologist measured the gastric antral cross-sectional area (CSA).

Each participant fasted from 22:00 the previous day and underwent ultrasound scanning in the right lateral position in the morning. We subsequently collected the fasting hunger and fasting glycaemia scores. Participants ingested 300 mL of clear water and were scanned every 15 min in the right lateral position. Scanning was suspended when the antrum returned to baseline or 120 min had passed. Participants subsequently ingested a 550 kcal meal (17) comprising chicken (150 g), pork (100 g), vegetables (100 g), rice (100 g), and 200 mL of clear water in 15 min. Scanning was performed at seven time points: 15, 30, 60, 90, 120, 180, and 240 min after meal ingestion, in the supine and right lateral positions. Participants were not permitted to lie down during the study.

### 2.3 Assessments

#### 2.3.1 Ultrasound scanning

**Figure 1:**
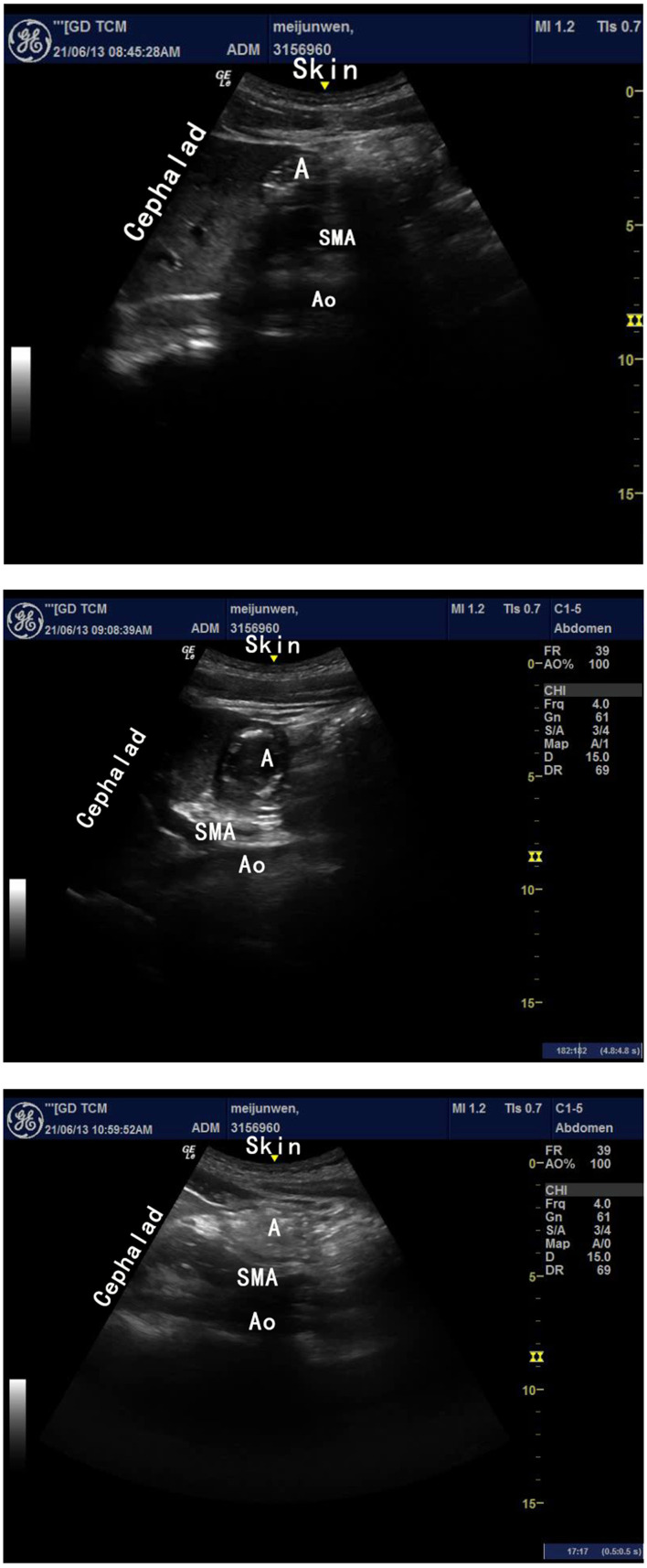
**(A–C)** Represent the ultrasound images of different gastric contents. **(A)** Empty, **(B)** Clear fluid, **(C)** Solid; SMA, superior mesenteric artery; Ao, aorta abdominalis; A, antrum of stomach.

Ultrasound measurements were acquired in the supine and right lateral positions using a low-frequency (2–5 MHz) curved array transducer (GE LOGIQ e NextGen; GE Healthcare, Boston, MA). The gastric antrum was located superficially in the epigastric area between the left lobe of the liver and the epigastrium in the sagittal or parasagittal scanning plane. Vascular landmarks included the aorta or inferior vena cava and superior mesenteric artery (Figures 1A–C). The antropyloric-duodenal transition was located in the axial plane with the pylorus on the right and the antrum on the left (21).

#### 2.3.2 Ultrasound measurement

The gastric antrum CSA in the right lateral position was determined using the following formula including two perpendicular diameters and the craniocaudal (CC) and anteroposterior (AP) diameters:


$$\begin{array}{l} CSA\;=\;\left(AP\;\times \;CC\;\times \;\pi \right)/4 \end{array}$$ (1)

We subsequently took the numerical average of the measurements recorded.

The GV was calculated using the formula described by Perlas et al. (22):


$$\begin{array}{r} GV\;=\;27.0\;+\;14.6\;\times \;CSA\;\left(right\;lateral\;position\right)\;1.28\\ \times \;age \end{array}$$ (2)

Based on our previous pilot study, the CSA 30 min and 240 min after a meal were used to calculate the 4 h GER (4HGER):


$$\begin{array}{r} 4HGER\;=\;\left[CSA\;\left(30\;\min\right)\;CSA\;\left(240\;\min\right)\right]/CSA\;\left(30\;\min\right)\\ \times \;100\% \end{array}$$ (3)

#### 2.3.3 Hunger score evaluation and glycaemia test

The hunger score was determined using a numeric rating scale (NRS) (0–10 points), with 0 corresponding to very hungry and 10 corresponding to not hungry at all (23). Venous blood samples were collected, and blood glucose concentrations were determined immediately using a portable blood glucose meter (Contour TS meter; Ascensia Inc., Basel, Switzerland).

### 2.4 Statistical analyses

#### 2.4.1 Statistics

All statistical analyses were performed using Excel 2019 (Microsoft Corporation, Redmond, WA, USA) and SPSS 26.0 (IBM SPSS, Armonk, NY, USA). Graphs were generated using Prism 9 (GraphPad, San Diego, CA, USA). A histogram was used to assess the data distribution. We conducted descriptive analyses of the demographic and clinical data. Continuous data are reported as mean (standard deviation, SD) for normal distributions and median (interquartile range) for non-normal distributions. Categorical data are presented as counts and percentages. The independent sample *t*-test or Mann–Whitney *U* test was used to evaluate the data between two groups. Categorical data were analyzed using Fisher's exact test. Fisher's exact test was preferred to the χ^2^ test on account of the small sample sizes.

Spearman correlation analysis was used to evaluate relationships between the CSA and hunger score. Stepwise multiple linear regression was used to assess the relationships between primary outcomes (emptying time of fluid, 4HGER) and other variables.

#### 2.4.2 Sample size

We calculated the necessary sample size based on our pilot study's (authors' unpublished data) outcome variables of 14 volunteers with a mean 4HGER of 30.68% (8.87%) in the older group and 45.91% (15.21%) in the younger comparison group. A sample size of 28 in each group would produce a power of 0.8 at an α of 0.05, using a two-tailed independent *t*-test. Eventually, we included 30 individuals each group in this clinical trial.

## 3 Results

### 3.1 Participant selection and baseline characteristics

Thirty healthy younger adults (Median age, 41.5 [31.8, 50.3] years; and BMI, 24.1[2.5] kg/m^2^) and 30 older adult participants (Median age, 70.8 [66.8, 74.0] years; and BMI, 24.8[2.5] kg/m^2^) were enrolled between April and July 2021. Distribution of sex was equal between the groups (39 women, 21 men). Fifty eight participants finished the study. One participant was excluded after they consumed fruit during the study, and another because they were not able to finish the meal in 15 min.

Age was significantly different between the groups (*P* < 0.001); yet no difference in BMI was observed. Age and BMI were analyzed using the Mann–Whitney *U* test and two independent *t*-tests, respectively (Table 1).

**Table 1:** Characteristics of the participants according to age, BMI, and sex.

**Demographics**	**Older**	**Younger**	***P* value**
Age, years, median (P_25_-P_75_)^a^	71.5 (68.6–73.0)	41.5 (31.8–50.3)	< 0.001^*^
BMI, kg/m^2^, mean (SD)^b^	24.8 (2.5)	24.1 (3.5)	0.575
Sex, female/total *n* (%)^c^	20/29 (69.0)	19/29(65.5)	0.789

^a^Mann–Whitney *U* test; ^b^Two independent *t*-tests; ^c^Fisher's Exact test.

^*^Significant difference between the two groups in age.

BMI, body mass index; SD, standard deviation.

### 3.2 Changes in fluid emptying time

Older participants showed longer fluid emptying times; however, no significant difference was observed in the median fluid emptying time between the younger (45 [30, 64] min; 95% confidence interval [CI]: 37, 60) and older (45 [30, 60] min; 95% CI: 38, 62) groups (*P* = 0.737).

The younger adult group exhibited a higher mean solid GER (0.51 [0.12]; 95% CI: 0.44, 0.58) than the older adult group (0.35 [0.09]; 95% CI: 0.30, 0.40) (*P* = 0.001) (Table 2).

**Table 2:** Fluid emptying time and 4 h gastric emptying rates.

**Components of gastric emptying**	**Older**	**Younger**	***t/z***	***P* value**
Fluid emptying time, min, median (P_25_, P_75_)^a^	45 (30, 60)	45 (30, 64)	−3.875	0.737
Four hour gastric emptying rate, mean (SD)^b^	0.35 (0.09)	0.51 (0.12)	−0.336	0.001

^a^Mann–Whitney *U* test; ^b^Two independent *t*-tests; SD, standard deviation.

### 3.3 Changes in blood glucose level, hunger NRS score, and gastric CSA

**Figure 2:**
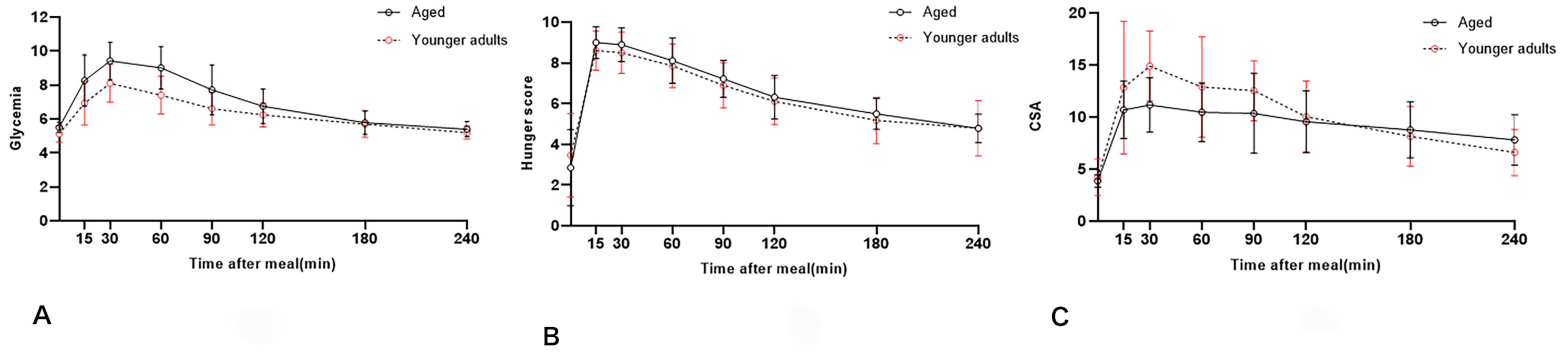
**(A–C)** Represent Changes in blood glucose level, hunger NRS score, and gastric CSA. The solid line represents the older adult group and the dashed line represents the younger adult group.

The mean blood glucose level at 0 min (older adults, 5.5 [0.3] vs. younger adults, 5.1 [0.5]; *P* < 0.05), 15 min (older adults, 8.3 [1.5] vs. younger adults, 6.9 [1.3]; *P* < 0.05), 30 min (older adults, 9.4 [1.1] vs. younger adults, 8.1 [1.1]; *P* < 0.01), and 60 min (older adults, 9.0 [1.2] vs. younger adults, 7.4 [1.1]; *P* = 0.01) differed significantly between the groups. No significant difference was observed in the hunger NRS score between groups at any time point. The mean gastric antrum CSA at 30 min differed significantly between the groups (older adults, 11.1 [3.1] vs. younger adults, 13.8 [3.6]; *P* < 0.05) (Figures 2A–C).

### 3.4 Correlation between hunger NRS score and gastric CSA

**Figure 3:**
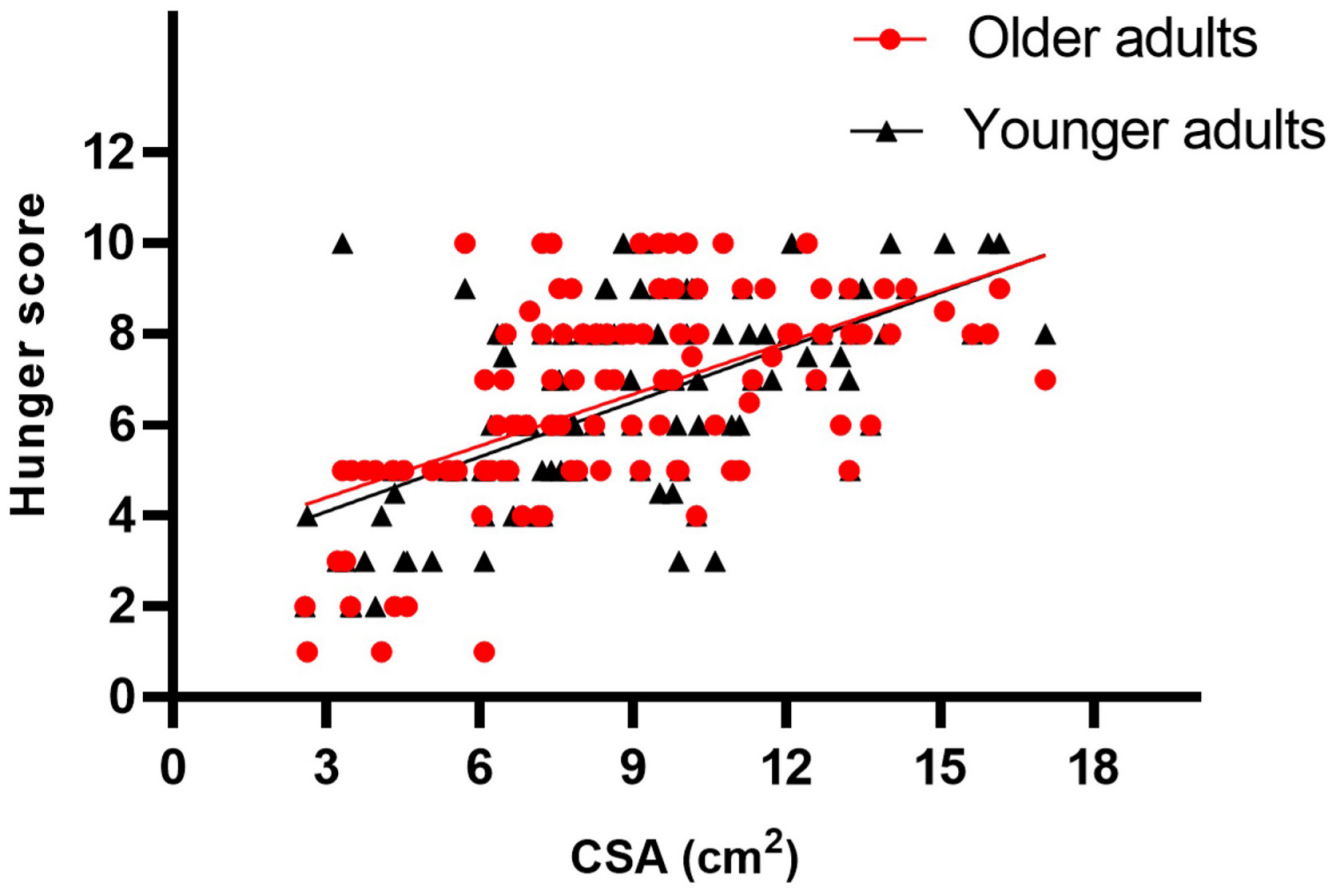
Represents the relationship between the gastric CSA and hunger NRS score. The gastric CSA and hunger NRS score showed a positive correlation (older adults, *r* = 5.44, *P* < 0.001; younger adults, *r* = 0.638, *P* < 0.001).

The gastric CSA and hunger NRS score showed a positive correlation (older adults, *r* = 5.44, *P* < 0.001; younger adults, *r* = 0.638, *P* < 0.001) (Figure 3).

### 3.5 Predictors of fluid emptying time and 4HGER

For fluid emptying time, stepwise linear regression analysis was conducted and included in the final model based on the *F*-value and *P* < 0.05 (*F* = 7.329, *P* = 0.003; *R* = 0.608, adjusted *R*^2^ = 0.319). A significant negative association was observed between BMI and fluid emptying time (β = −0.562; *P* = 0.002), and a significant positive association was observed between age and fluid emptying time (β = 0.347; *P* = 0.041).

For 4HGER, all independent variables (age, sex, BMI, fasting CSA, glycaemia, and hunger NRS score) in the initial model were included in the stepwise regression and removed one by one based on the criteria of *P* < 0.05 for inclusion in the final model (*F* = 15.137, *P* < 0.001; *R* = 0.740, adjusted *R*^2^ = 0.512). A significant negative association was observed between BMI and 4HGER (β = −0.022; *P* = 0.001). A significant positive association was observed between age and 4HGER (β = −0.003; *P* = 0.002). Based on the stepwise linear regression analysis conducted in this study, the following predictive models were developed to estimate Fluid emptying time (FE-time) and the 4 h gastric emptying rate (4HGER) in adults:


$$\begin{array}{l} FE\;-\;time\;=\;120.642\;-\;0.562\;\times \;BMI\;+\;0.347\;\times \;Age \end{array}$$ (4)

For the 4 h gastric emptying rate (4HGER), the regression equation was:


$$\begin{array}{l} 4HGER=1.144\;-\;0.022\;\times \;BMI\;-\;0.003\;\times \;Age \end{array}$$ (5)

## 4 Discussion

This prospective study utilized ultrasound to evaluate the gastric emptying time of fluid and gastric emptying rate (GER) of rice-based solid meals in older and younger adult volunteers. To our knowledge, this is the first study to report the GER of rice meals in older adults using ultrasonography. Current preoperative fasting guidelines recommend standardized durations for adults (8 h for solids, 6 h for starchy liquids, and 2 h for clear fluids), without a specific focus on age-related physiological variations. Age-associated declines in gastrointestinal motility may prolong gastric emptying, suggesting that existing protocols require critical reassessment. Our hypothesis that GER decreases with advancing age in older adults was supported by the findings, showing that the average GER in older adults (0.35 [0.09]) is significantly lower than that of younger adults (0.51 [0.12]). However, the liquid emptying time did not vary by age.

Gastric emptying, influenced by factors such as BMI, sex, age, and meal composition, has been extensively studied. While fasting guidelines uniformly apply to adults regardless of age [unlike pediatric protocols (24)], accumulating evidence indicates delayed GE in older populations. For instance, Brogna et al. (25) reported significantly prolonged solid-phase GE in older adults (mean age: 75 years) vs. younger adults (mean age: 30 years), though total gastrointestinal transit times remained comparable. Radionuclide assessments further corroborate reduced GER in older adults, potentially attributable to autonomic dysfunction and diminished secretory capacity (26). Conversely, other studies report only modest GE slowing in healthy aging, with changes comparable to younger cohorts (27). Discrepancies persist, however, as some investigations—including a model-based meta-analysis spanning 66 studies—found no age-dependent GE alterations, though limited data from older adults were included^11^.

Mechanistically, gastric emptying (GE) involves coordinated motility of the proximal and distal stomach, antrum, pylorus, and duodenum, regulated by neural and humoral responses to nutrient exposure. The emptying patterns of liquids and solids differ fundamentally: solids are ground from large particles into small ones, whereas the GE of non-caloric liquids is driven primarily by gravity and volume (28). Our results align with this paradigm, revealing preserved liquid emptying kinetics across age groups, contrasting with marked solid-phase gastric emptying rate (GER) differences.

The pathophysiology of age-related GE delay remains debated but likely involves multifactorial mechanisms. Healthy aging is characterized by gradual declines in homeostatic regulation, including autonomic nervous system degeneration and glucose dysregulation (29, 30). Notably, older adults exhibited elevated postprandial glucose levels in this study, a phenomenon potentially linked to delayed GE through glucoregulatory feedback loops. This interaction warrants particular attention in elderly diabetic patients, who face heightened risks of perioperative glycemic instability due to stress-induced hormonal fluctuations.

In the present study, the blood glucose levels after meals differed significantly between younger and older adults. Older participants exhibited a larger increase in postprandial glucose levels, which returned to those comparable to the levels in younger adults after 90 min. This transient hyperglycemia in older adults—despite elevated fasting glucose—suggests a potential link between delayed gastric emptying (GE) and impaired glucoregulatory responses. Mechanistically, rapid postprandial glucose spikes may activate inhibitory feedback mechanisms (e.g., via the ileal brake or incretin pathways), further slowing GE in aging populations (12). Notably, Watson et al. (12) reported paradoxical GE patterns: older non-diabetic individuals exhibited slower solid-phase emptying than younger counterparts, whereas patients with well-controlled type 2 diabetes demonstrated accelerated GE compared to age- and BMI-matched controls. These findings underscore the bidirectional relationship between glycaemia and GE, wherein hyperglycemia suppresses motility (as shown by Fraser et al. (31) in type 1 diabetes), while hypoglycemia enhances it (7). The pronounced glucose fluctuations observed in older adults highlight the clinical urgency of refining preoperative fasting protocols. Prolonged or inappropriate fasting may exacerbate perioperative glycemic instability, particularly in elderly patients with prediabetes or undiagnosed metabolic dysfunction. Current guidelines, which prioritize aspiration risk mitigation, often overlook the metabolic consequences of prolonged fasting, such as reactive hypoglycemia or ketosis. Future research should focus on age-specific fasting strategies that balance gastric emptying kinetics with metabolic stability. For example, shorter fasting intervals for solids (e.g., 6–8 h) combined with carbohydrate-rich clear fluids up to 2 h preoperatively could mitigate both aspiration risk and glycemic extremes. Additionally, continuous glucose monitoring in perioperative studies may elucidate optimal fasting durations tailored to individual metabolic profiles.

Multiple modalities are available for assessing gastric contents, including magnetic resonance imaging (MRI), ^13^C breath tests, and gastric ultrasonography—all validated for clinical and research applications. However, accessibility to advanced imaging technologies varies across healthcare settings, limiting their universal utility. This raises the question of whether subjective indicators, such as hunger perception, could complement objective assessments. While hunger serves as a physiological marker of energy depletion and is widely used to infer gastric emptying status, our findings revealed a paradoxical dissociation: despite a positive correlation between hunger scores (NRS) and gastric antral cross-sectional area (CSA), hunger scores returned to baseline within 4 h postprandially, even when residual gastric volume (GV) remained substantial (approximately 30%). This discrepancy aligns with prior reports demonstrating that self-reported hunger NRS scores poorly predict residual fluid volumes (23, 32). For instance, Qiu et al. (23) concluded that while hunger scores may aid in estimating preoperative fasting adequacy, they lack precision in quantifying gastric content volumes. Similarly, pediatric studies caution against relying on thirst or hunger ratings to gauge gastric residue (32). Age-related declines in visceral sensitivity further complicate this issue. Healthy older adults exhibit attenuated perception of gastric distension (33), likely due to blunted afferent signaling. Consequently, hunger scores may reflect subjective satiety but fail to reliably indicate fasting-state gastric contents, particularly in elderly populations. These limitations underscore the necessity of integrating objective measurements (e.g., ultrasound) with clinical judgment, rather than relying solely on subjective cues.

A critical question arises: Can subjective hunger perception serve as a surrogate marker for glycemic instability induced by prolonged fasting in older adults? As illustrated in Figure 2, despite blood glucose levels returning to baseline within 4 h postprandially in both groups, gastric emptying rates (GER) remained incomplete (≈50% residual volume), indicating discordance between glycemic recovery and gastric content clearance. Notably, hunger scores failed to parallel the rapid normalization of blood glucose at 240 min, further underscoring the disconnection between perceived hunger and metabolic state. These findings suggest that neither blood glucose levels nor hunger scores reliably predict residual gastric volume (GV), particularly in older adults with age-related blunting of visceral afferent signaling (33, 34).

With the global aging population, more focus should be placed on the perioperative management of older patients. Asia has the largest population of older individuals; therefore, GE studies conducted in Asia should consider our findings. Slow GE can lead to differences in the assessment of fasting, increasing the incidence of complications, such as aspiration. However, according to the ASA guidelines (1), perioperative fasting time is determined by the type of food, not BMI or age. Herein, we found a relationship between age, BMI, and GE. Recently, fasting time has been reduced to enhance recovery after surgery. Prolonged fasting does not mean that more research is necessary before the next guideline can be formulated. Recent research on gastric emptying (GE) challenges existing restrictive fasting durations (19, 35) and suggests avoiding prolonged fasting (18, 24). However, overly conservative fasting times may also increase the risk of aspiration in certain populations. Elderly individuals are more sensitive to fasting and often experience complications such as hypotension and hypoglycemia, which heighten perioperative risks.

This study has several limitations that warrant consideration. First, gastric emptying (GE) was assessed for a maximum of 240 min postprandially, precluding the determination of complete emptying times—particularly in older adults, where delayed GE may persist beyond this window. While differences were inferred from 4 h emptying ratios, this truncated observation period risks underestimating age-related delays. Second, the exclusion of individuals with obesity (BMI >30 kg/m^2^) limits the generalizability of findings to a population increasingly prevalent in clinical practice. Notably, conflicting evidence exists regarding BMI's role in GE: Jackson et al. (36) reported delayed GE in obese women (BMI = 34.5 kg/m^2^) vs. lean controls using ^13^C-octanoic acid breath tests, whereas Buchholz et al. (37) found no BMI-GE association in cohorts with lower BMI disparities (26 vs. 45 kg/m^2^). This discrepancy may stem from demographic imbalances (e.g., obese cohorts dominated by younger women vs. non-obese older men), where age and sex confounders complicate interpretation. Third, the standardized 15 min meal consumption period may not account for age-related variations in mastication efficiency. Prolonged chewing in older adults could theoretically alter particle size and antral processing time, potentially biasing GE measurements. Future studies should extend observation periods, include obese cohorts, and incorporate mastication time as a covariate to refine preoperative fasting guidelines.

## 5 Conclusion

GE, assessed using ultrasound after consumption of rice-based meals, was significantly delayed in older adults compared with that in younger adults. The estimated GER decreased as the age and BMI of participants increased. Our findings have implications for medical professionals who manage patients under anesthesia and may help reduce adverse outcomes.

## Data Availability

The original contributions presented in the study are included in the article/supplementary material, further inquiries can be directed to the corresponding author.
